# Multiple in-hospital counseling increases six-month smoking abstinence among individuals participating in a hospital-initiated smoking cessation program

**DOI:** 10.1186/s13722-022-00310-9

**Published:** 2022-05-21

**Authors:** Chin-Wei Kuo, Chuan-Yu Chen, Chih-Hsing Wu, Chang-Wen Chen, Fei-Ran Guo, Szu-Chun Yang

**Affiliations:** 1 Division of Pulmonary Medicine, Department of Internal Medicine, National Cheng Kung University Hospital, College of Medicine, National Cheng Kung University, Tainan, Taiwan; 2 Institute of Clinical Medicine, College of Medicine, National Cheng Kung University, Tainan, Taiwan; 3 Department of Family Medicine, National Cheng Kung University Hospital, College of Medicine, National Cheng Kung University, Tainan, Taiwan; 4 Health Promotion Association, National Cheng Kung University Hospital, College of Medicine, National Cheng Kung University, Tainan, Taiwan; 5 Department of Family Medicine, National Taiwan University Hospital, Taipei, Taiwan; 6 Department of Family Medicine, College of Medicine, National Taiwan University, Taipei, Taiwan

**Keywords:** Hospitalized smoker, Smoking cessation, Cessation counseling

## Abstract

**Background:**

A cessation program for hospitalized smokers is an effective strategy to achieve smoking abstinence. The effects of multiple in-hospital counseling sessions on 6-month smoking abstinence require further investigation.

**Methods:**

We retrospectively analyzed the data of smokers who participated in hospital-initiated cessation programs at a medical center between 2017 and 2019. Data on age, sex, comorbidities, daily number of cigarettes, cessation motivation, nicotine dependence, cessation medications, discharge diagnosis, length of hospitalization, and intensive care unit admission were collected. We conducted multiple logistic regression analysis to investigate the effect of multiple in-hospital counseling sessions on 6-month sustained smoking abstinence. Sensitivity analyses were carried out excluding participants who underwent post-discharge cessation programs and assuming that the loss to follow-up participants had failure in 6-month smoking abstinence.

**Results:**

A total of 1943 participants aged ≥ 20 years were analyzed. Compared with single in-hospital counseling session, the adjusted odds ratios (ORs) for 2 and ≥ 3 counseling sessions were 1.44 (95% confidence interval [CI] 1.05 to 1.98) and 2.02 (95% CI 1.27 to 3.22), respectively, with a significant trend for increasing the number of counseling sessions (*P* < 0.001). The results remained significant after excluding participants who underwent a post-discharge cessation program or when assuming that lost to follow-up participants had failure in smoking abstinence.

**Conclusion:**

Multiple in-hospital counseling sessions were associated with a higher 6-month sustained smoking abstinence rate. This strategy could be used to reduce the prevalence of smoking.

## Introduction

Cigarette smoking contributes to a variety of diseases and results in premature disabilities, which in turn increases healthcare expenditures. According to a global report, the medical cost of smoking-attributable diseases was 422 billion US dollars, which was approximately 5.7% of global healthcare expenditures in 2012 [1]. Compared with individuals who have never smoked, current smokers have a significant reduction in life expectancy [2]. Cessation of smoking can significantly reduce excess morbidity and mortality resulting from cigarette smoking [3]. A good smoking cessation program includes both pharmacotherapy and counseling. Cessation counseling is usually provided by physicians, nurses, or trained counsellors in a face-to-face manner or using a communication device [4]. The Taiwan Bureau of Health Promotion initiated nationwide smoking cessation programs in 2002 [5]. This department grants subsidies to institutions that carry out the program and provides well-regulated registered data.

Smoking cessation during hospitalization is one of the best ways to conduct cessation programs. Hospitalized smokers personally experience smoking-attributable diseases, which, in turn, increase their motivation for abstinence [6, 7]. Hospitals are non-smoking areas, and acute illness results in a certain degree of disability for smokers, reducing their ability to access tobacco products [6, 7]. The effectiveness of hospital-initiated smoking cessation programs has been well reported in previous studies [8]. Intensive cessation counseling during hospitalization increases smoking cessation rates [9]. A previous meta-analysis showed that multiple counseling sessions produce higher abstinence rates than zero or one session, but most of the studies enrolled were conducted in outpatient services [10]. To date, the effect of multiple in-hospital cessation counseling sessions on smoking abstinence has not yet been explored in Taiwan. We hypothesized that multiple cessation counseling sessions would increase 6-month smoking abstinence in individuals participating in hospital-initiated cessation programs. We attempted to verify this hypothesis using well-registered data from a medical center in southern Taiwan.

## Methods

### Study design

We retrospectively analyzed the data of smokers who participated in a hospital-initiated cessation program at a medical center in southern Taiwan. The in-hospital cessation program adopted the ‘Ottawa model,’ a systematic approach for smoking cessation delivered within healthcare institutes that includes identifying the smoking history and status of participants, providing brief cessation counseling and pharmacotherapy and arranging post-discharge follow-up support [11]. A telephone interview was used to determine sustained smoking abstinence at the sixth month after the first counseling session. Participants aged ≥ 20 years who first entered the program from 1 January 2017 to 30 November 2019 were included in the analysis. We further excluded individuals who were censored within the 6-month follow-up period. This study was approved by the institutional review board of National Cheng Kung University Hospital before commencement (B-ER-110-126).

### In-hospital cessation program and data collection

The smoking cessation team at National Cheng Kung University Hospital consisted of two full-time government-certified counselors and three doctors specializing in family or internal medicine. In the first counseling session, counselors recorded the basic information of the participants, educated the participants on the harms of tobacco, motivated smoking cessation, and instructed the participants to manage nicotine withdrawal symptoms. Specifically, counselors collected data of individual demographic characteristics and comorbidities, including cardiovascular disease, cancer, cerebrovascular disease, diabetes mellitus, hypertension, liver disease, respiratory system disease, and renal disease. Smoking history, including the number of cigarettes smoked daily, was also recorded. Nicotine dependence was assessed using the Fagerstrom test for nicotine dependence (FTND) [12]. Individuals with an FTND score of 4 or more were recommended to receive pharmacological smoking-cessation medications, including nicotine replacement therapy (NRT) in the form of patches, inhalers, or gum. Alternative medications were non-NRTs, including varenicline and bupropion. Participants with an FTND score of 7 or more were allowed to use NRT and non-NRT concomitantly. Cost reduction for smoking cessation medications was granted according to the Bureau of Health Promotion guidelines [5]. Counselors evaluated each individual’s motivation, which could be self-motivated or motivated by family members, colleagues, or medical staffs. In addition, information about the harmful effects of tobacco and management of withdrawal symptoms was provided. To increase the likelihood of smoking abstinence, counselors were requested to provide two or more in-hospital counseling sessions with permission from the participants. In the second and subsequent counseling sessions, counselors reinforced the participants’ determination to quit smoking, management of withdrawal symptoms, and adherence to cessation medications. Each counseling session lasted for approximately 15–20 min. A post-discharge cessation appointment was arranged for each patient by the outpatient department. By reviewing the electronic medical records of these patients, we determined the main discharge diagnosis, length of hospitalization, and intensive care unit (ICU) admission.

### Statistical analysis

The characteristics of the hospitalized smokers are presented as numbers and percentages. We compared the characteristics of individuals who were and were not successful in achieving 6-month smoking abstinence using the chi-square test. The main analysis using multiple logistic regression was conducted to investigate the effects of multiple in-hospital counseling sessions on 6-month smoking abstinence after controlling for age (≥ 65 versus < 65), sex (male versus female), number of comorbidities (≥ 2 versus < 2), number of cigarettes smoked daily (> 20 versus ≤ 20), FTND score (≥ 7 and 4–6 versus < 4), motivation (self-motivated versus not self-motivated), main discharge diagnosis (coronary artery disease [CAD], heart failure [HF], chronic obstructive pulmonary disease [COPD], pneumonia, or cancer versus without) [13], length of hospital stay (> 4 days versus ≤ 4 days), ICU admission (yes versus no), and cessation medications (NRT, non-NRT, and both versus no). Associations were expressed as odds ratios (ORs) and 95% confidence intervals (CIs). To assess the trends between increasing the number of counseling sessions and successful smoking abstinence, the *F* test for linear trend of the adjusted ORs were estimated. All *P-values* were two-sided, and a value of less than 0.05 was considered statistically significant. SAS software (version 9.4; SAS Institute, Cary, NC, USA) was used to perform the analyses.

### Sensitivity analyses

Sensitivity analyses were performed to test the robustness of the results. We first assumed that all participants who were censored failed to achieve 6-month smoking abstinence. We also excluded participants undergoing outpatient cessation counseling and medication prescriptions from the sensitivity analysis.

## Results

A total of 2900 individuals who participated in the hospital-initiated smoking cessation program were included in the analysis. Among them, 957 were censored after the commence of program, leaving the data of 1943 participants for the main analysis. Table 1 shows the demographic and clinical characteristics of the participants who were censored and those in the main analysis. Participants who successfully achieved 6-month smoking abstinence were older, had more comorbidities, less daily cigarette consumption, and lower FTND scores than those who failed to achieve 6-month smoking abstinence. They were more likely to be admitted for cancer, CAD/HF, COPD/pneumonia and encountered ICU admission or hospitalization for > 4 days. Individuals who were successful in achieving 6-month smoking abstinence had more in-hospital counseling than those who were unsuccessful (*P* = 0.001). There were no significant differences in sex, self-motivation, or cessation medications between the two groups. The 6-month smoking abstinence rates were 38.2%, 47.1%, and 52.3% for individuals who received one, two, three, or more counseling sessions, respectively.

**Table 1:** Characteristics of smokers participating in a hospital-initiated cessation program

	Participants who censored (*n* = 957)	Participants for analysis (*n* = 1943)		
		6-month smoking abstinence		
		Success (*n* = 778)	Failure (*n* = 1165)	*P*
Age (years)				< 0.001
< 65	789 (82.4)	556 (71.5)	981 (84.2)	
≥ 65	168 (17.6)	222 (28.5)	184 (15.8)	
Sex				0.237
Male	886 (92.6)	716 (92.0)	1054 (90.5)	
Female	71 (7.4)	62 (8.0)	111 (9.5)	
Comorbidities^a^				< 0.001
< 2	631 (65.9)	405 (52.1)	729 (62.6)	
≥ 2	326 (34.1)	373 (47.9)	436 (37.5)	
Daily cigarette smoking				0.011
≤ 20	275 (28.7)	311 (40.0)	400 (34.3)	
> 20	551 (57.6)	467 (60.0)	765 (65.7)	
Missing	131 (13.7)	0 (0.0)	0 (0.0)	
FTND				< 0.001
< 4	90 (9.4)	109 (14.0)	82 (7.0)	
4 to 6	472 (49.3)	411 (52.8)	527 (45.2)	
≥ 7	395 (41.3)	258 (33.2)	556 (47.7)	
Motivation				0.769
Self-motivated	326 (34.1)	207 (26.6)	303 (26.0)	
Not self-motivated	631 (65.9)	571 (73.4)	862 (74.0)	
Discharge diagnosis				< 0.001
Cancer	195 (20.4)	157 (20.2)	158 (13.6)	
CAD/HF	165 (17.2)	212 (27.3)	207 (17.8)	
COPD/pneumonia	22 (2.3)	37 (4.8)	40 (3.4)	
Others	575 (60.1)	372 (47.8)	760 (65.2)	
ICU admission				< 0.001
Yes	213 (22.3)	251 (32.3)	156 (13.4)	
No	744 (77.7)	527 (67.7)	1009 (86.6)	
Hospitalization				< 0.001
≤ 4 days	456 (47.6)	336 (43.2)	637 (54.7)	
> 4 days	501 (52.4)	442 (56.8)	528 (45.3)	
Cessation medications				0.175
No use	823 (86.0)	673 (86.5)	1020 (87.6)	
NRT	75 (7.8)	56 (7.2)	81 (7.0)	
Non-NRT	22 (2.3)	31 (4.0)	28 (2.4)	
NRT and non-NRT	37 (3.9)	18 (2.3)	36 (3.1)	
Number of in-hospital counseling				0.001
1	773 (80.8)	616 (79.2)	995 (85.4)	
2	113 (11.8)	104 (13.4)	117 (10.0)	
≥ 3	71 (7.4)	58 (7.5)	53 (4.6)	

*CAD* coronary artery disease, *COPD* chronic obstructive pulmonary disease, *FTND* Fagerstrom test for nicotine dependence, *HF* heart failure, *ICU* intensive care unit, *NRT* nicotine replacement therapy

^a^Comorbidities include cardiovascular disease, cancer, cerebral vascular disease, diabetes mellitus, hypertension, liver disease, respiratory system disease, and renal disease

### Predictors of 6-month smoking abstinence

Multiple logistic regression analyses of predictors of successful 6-month smoking cessation are shown in Table 2. The adjusted ORs for participants undergoing 2 and ≥ 3 in-hospital counseling sessions were 1.44 (95% CI 1.05 to 1.98) and 2.02 (95% CI 1.27 to 3.22), respectively. There was a trend between the number of in-hospital counseling sessions and successful 6-month smoking abstinence (*P* < 0.001). Age ≥ 65 years, ≥ 2 comorbidities, hospitalization for cancer or CAD/HF, ICU admission, hospitalization > 4 days were associated with an increased success rate. Patients with higher FTND scores were less likely to achieve 6-month smoking abstinence.

**Table 2:** Multiple logistic regression analyses

Covariables^a^	Main analysis (*n* = 1943)			Censored participants (*n* = 2900)			Excluding participants with out-patient cessation (*n* = 1834)		
	Adjusted OR (95% CI)	*P*	*P* for trend	Adjusted OR (95% CI)	*P*	*P* for trend	Adjusted OR (95% CI)	*P*	*P* for trend
Age ≥ 65 years	1.78 (1.40–2.28)	< 0.001		1.29 (0.98–1.69)	0.073		1.72 (1.34–2.20)	< 0.001	
Male	1.04 (0.73–1.49)	0.839		1.10 (0.79–1.51)	0.579		1.02 (0.71–1.46)	0.920	
≥ 2 comorbidities^b^	1.25 (1.01–1.53)	0.037		1.17 (0.97–1.41)	0.099		1.27 (1.03–1.58)	0.026	
Daily cigarette smoking > 20	1.09 (0.86–1.38)	0.487		0.75 (0.62–0.90)	0.003		1.09 (0.85–1.39)	0.492	
FTND									
< 4	Reference			Reference			Reference		
4 to 6	0.49 (0.34–0.69)	< 0.001		0.69 (0.47–1.01)	0.056		0.45 (0.32–0.64)	< 0.001	
≥ 7	0.28 (0.19–0.42)	< 0.001		0.50 (0.30–0.84)	0.009		0.27 (0.18–0.41)	< 0.001	
Self-motivated	1.19 (0.95–1.50)	0.139		0.67 (0.49–0.91)	0.012		1.20 (0.96–1.54)	0.098	
Discharge diagnosis									
Others	Reference			Reference			Reference		
Cancer	2.18 (1.66–2.86)	< 0.001		1.67 (1.32–2.13)	< 0.001		2.19 (1.67–2.87)	< 0.001	
CAD/HF	1.40 (1.04–1.88)	0.028		1.24 (0.95–1.62)	0.110		1.35 (0.99–1.85)	0.058	
COPD/pneumonia	1.58 (0.96–2.61)	0.073		1.32 (0.82–2.12)	0.252		1.45 (0.83–2.56)	0.195	
ICU admission	2.82 (2.14–3.73)	< 0.001		2.41 (1.89–3.06)	< 0.001		2.84 (2.13–3.78)	< 0.001	
Hospitalization > 4 days	1.43 (1.15–1.77)	0.001		1.19 (0.98–1.45)	0.076		1.46 (1.17–1.82)	0.001	
Cessation medications									
No use	Reference			Reference			Reference		
NRT	1.12 (0.74–1.68)	0.599		1.05 (0.73–1.52)	0.784		1.10 (0.70–1.73)	0.684	
Non-NRT	1.60 (0.89–2.86)	0.115		1.42 (0.85–2.38)	0.180		1.83 (0.93–3.59)	0.081	
NRT and non-NRT	0.80 (0.42–1.51)	0.484		0.81 (0.46–1.45)	0.485		0.97 (0.48–1.97)	0.927	
Number of in-hospital counseling			< 0.001			0.010			0.012
1	Reference			Reference			Reference		
2	1.44 (1.05–1.98)	0.025		1.21 (0.92–1.61)	0.180		1.43 (1.03–1.98)	0.034	
≥ 3	2.02 (1.27–3.22)	0.003		1.82 (1.21–2.73)	0.004		1.62 (0.98–2.66)	0.059	

*CAD* coronary artery disease, *CI* confidence interval, *COPD* chronic obstructive pulmonary disease, *FTND *Fagerstrom test for nicotine dependence, *HF *heart failure, *ICU *intensive care unit, *NRT *nicotine replacement therapy, *OR* odds ratio

^a^References for covariables: age (≥ 65 versus < 65), sex (male versus female), number of comorbidities (≥ 2 versus < 2), number of cigarettes smoked daily (> 20 versus ≤ 20), FTND score (≥ 7 and 4–6 versus < 4), motivation (self-motivated versus not self-motivated), main discharge diagnosis (cancer, CAD/HF, COPD/pneumonia versus without), ICU admission (yes versus no), length of hospital stay (> 4 days versus ≤ 4 days), and cessation medications (NRT, non-NRT, and both versus no)

^b^Comorbidities include cardiovascular disease, cancer, cerebral vascular disease, diabetes mellitus, hypertension, liver disease, respiratory system disease, and renal disease

### Sensitivity analyses

The results of the sensitivity analysis are listed in Table 2. Assuming censored participants failed to abstain, adjusted ORs for participants who received 2 and ≥ 3 in-hospital counseling were 1.21 (95% CI 0.92 to 1.61) and 1.82 (95% CI 1.21 to 2.73), respectively. There was a trend between the number of in-hospital counseling sessions and successful 6-month smoking abstinence (*P* = 0.010). After excluding individuals participating in outpatient cessation programs, the adjusted ORs for participants who received 2 and ≥ 3 in-hospital counseling sessions were 1.43 (95% CI 1.03 to 1.98) and 1.62 (95% CI 0.98 to 2.66). There was a trend between the number of in-hospital counseling sessions and successful 6-month smoking abstinence (*P* = 0.012).

## Discussion

Previous studies have found that different diseases in participants, levels of intention to quit, and degrees of nicotine dependency interfere with the abstinence rate related to hospital-initiated smoking cessation programs [14, 15]. However, to date there have been no studies investigating the effect of multiple in-hospital counseling sessions on sustained smoking abstinence in Taiwan. In this hospital cohort, we retrospectively analyzed data from 1943 participants who participated in a hospital-initiated smoking cessation program. Because the collected items (Table 1) including comorbidities, number of cigarettes smoked daily, the FTND scores, and cessation medications are strictly mandated by the Bureau of Health Promotion, the information was considered credible. By using a multiple logistic regression model to control for age, sex, ICU admission, main discharge diagnosis, length of hospitalization, and these factors, we found that multiple in-hospital counseling sessions were associated with a higher rate of 6-month smoking abstinence, and a significant trend related to an increased number of counseling sessions was observed (Table 2). The additional sensitivity analyses further proved the robustness of our results. We thus concluded that multiple counseling sessions may help increase 6-month smoking abstinence in individuals participating in hospital-initiated cessation programs in Taiwan.

A possible explanation for improvements in the 6-month smoking abstinence with multiple in-hospital cessation counseling sessions is that multiple counseling sessions provide a sufficient educational intensity and avoid excessive work in each counseling sessions. Multiple cessation counseling sessions increased the abstinence rate of smokers who participated in the outpatient cessation programs [10]. This is likely to be true for hospitalized smokers who have experienced the effects of smoking-related diseases. In addition, although attending outpatient smoking cessation services can increase the counseling intensity, it might be better to provide sufficient counseling intensity during hospitalization because smokers do not always return to the outpatient department after discharge.

Most participants received one counseling session in our study. Potential barriers for multiple cessation counseling include few staff members, a short hospital stay, and time conflict with the examination or procedure. Creating a computer system that allows counselors to find the best time slots for visiting the patients merits future work.

In our multiple regression analysis, older age, multiple comorbidities, and lower FTND scores were associated with a higher smoking abstinence rate. These results are consistent with those of previous studies [14, 15]. Another investigation found that awareness of the association between smoking and ICU admission increases 6-month smoking cessation rates, [16] which corroborates our finding that ICU admission is a significant predictor of smoking abstinence. In the present study, we did not observe any positive association between smoking cessation medications and sustained smoking cessation. Plausible reasons for this result include the small proportion of participants receiving medications. Our cohort consisted of patients with different diseases, for which the effects of cessation medications were reported to be different [17, 18].

Our study has several limitations. First, the 6-month smoking abstinence of the participants was assessed by telephone interviews without biochemical confirmation. Second, counselors self-determined who would be provided with two or more in-hospital counseling sessions. Hospitalized smokers who were deeply motivated were more likely to be selected by counselors for multiple counseling sessions. Although we did not quantify individual motivation using the Motivation to Stop Scale, [19] self-motivation was included as one of the confounders in the multiple regression analysis. Consequently, the results are not biased. The length of hospital stay also interfered with the selection of multiple counseling. However, because the trend for an increasing number of counseling sessions remained significant after adding it as a covariate, the validity of our study would not be threatened. Third, many in-hospital participants did not undergo post-discharge cessation because of co-payment issues; therefore, adherence to cessation medications might be concerning. Nevertheless, our 6-month smoking abstinence rate was similar to that for hospitalized smokers, [20] and the sensitivity analysis excluding participants undergoing post-discharge cessation showed similar results; therefore, the effect of multiple in-hospital counseling sessions on smoking abstinence is believed to be valid. Fourth, this study was conducted at a single medical center in Taiwan, and the results should be generalized with caution. Finally, 957 participants who were censored were excluded from the main analysis; thus, the ORs were overestimated. However, a sensitivity analysis assuming that all participants who were censored failed to achieve 6-month smoking abstinence was performed. The effect of multiple in-hospital counseling sessions on 6-month smoking abstinence was still observed.

In conclusion, multiple in-hospital counseling sessions were associated with a higher rate of 6-month smoking abstinence. This strategy could be applied in smoking programs to reduce the prevalence of smoking.

## Data Availability

All the data in this study are available to others if the data intend to be used for scholarly purposes such as a systematic review. The data are not publicly available because the use of the National Health Insurance Research Database is limited to research purposes only.

## Abbreviations

CAD: Coronary artery disease
CI: Confidence interval
COPD: Chronic obstructive pulmonary disease
FTND: Fagerstrom Test for Nicotine Dependence
HF: Heart failure
ICU: Intensive care unit
NRT: Nicotine replacement therapy
OR: Odds ratios
